# Literary Notes

**Published:** 1906-08

**Authors:** 


### LITERARY NOTES.

"Diet After Weaning" is the title of a handsome brochure issued by the Mellin's Food Company, Boston. It is a duodecimo of forty-six pages, giving useful hints for baby-feeding and is illustrated by numerous half-tones of fine healthy looking children. It is printed on heavy book paper with illuminated margins and is bound in red vellum, making a beautiful specimen of the bookmakers art. It will be sent free on application to the publishers by any mother or physician, on mentioning this Journal.

Messrs P. Blakiston's Son and Company, Philadelphia, announce that the second edition of the treatise on Diseases of Children, by J. Madison Taylor and William H. Wells, has been translated into Italian by Dr. Mario Flamimi, of the Pediatric Clinic of Rome, with contributions by Professor Concetti and Dr. Valagussa. This is a distinctive honor few American books have been accorded and the success of the work abroad is only second to the favor it has received at home.

